# An Investigation of the Effects of Yoga on the Psychological Well-Being of Young Athletes: A Randomized Controlled Trial

**DOI:** 10.7759/cureus.85452

**Published:** 2025-06-06

**Authors:** Priyanka Saraswati, Satish Kanaujia, Ghanshyam Singh Thakur, Bhuwan Chandra Kapri

**Affiliations:** 1 Humanistic Studies, Indian Institute of Technology (Banaras Hindu University) Varanasi, Varanasi, IND; 2 Naturopathy and Yoga, Hemvati Nandan Bahuguna Garhwal University, Srinagar, IND; 3 Physical Education, Banaras Hindu University, Varanasi, IND

**Keywords:** awareness, mindfulness, psychological flexibility, stress, yoga

## Abstract

Background

Yoga and mind-body mindfulness exercises emphasize awareness-building to help athletes perform better physically and psychologically under trying circumstances. The purpose of the study was to assess how yoga practice affects athletes' stress levels, levels of mindfulness, and psychological flexibility.

Methods

The design of the study was a randomized controlled trial. A total of 88 participants were randomly assigned to two groups in a 1:1 ratio: the yoga practice group and the control group. The yoga group received the yogic intervention, comprising a planned practice of pranayama (breath control) and meditation over a month, which started with a customary prayer. To improve physical vitality, mental clarity, and emotional balance, the practice included a variety of breathing techniques, including *Kapal Bhati*, *Ujjai*, *Bhastrika*, *Sitli*, *Sitkari*, *Anulom Vilom*, and *Bhramari*. The control group didn't receive any treatment. Recreational athletes, aged 18 to 45, were screened and enrolled. Participants received a 30-day intervention, and information on athletes' stress levels, psychological flexibility, and mindfulness was gathered.

Results

Following the intervention, the participants' stress levels were found to be significantly lower (p < 0.001). Those who had participated in the yoga intervention reported fewer negative emotional states and higher dispositional mindfulness levels (p < 0.001). The athletes' everyday psychological flexibility skills could be strongly predicted by stress reduction (adjusted R^2 ^= 0.660, p = 0.000) and mindfulness improvement (adjusted R^2 ^= 0.581, p = 0.000).

Conclusion

Beyond just improving physical health, yoga has many positive effects on mental and emotional health, especially for athletes. Regular use of yoga practices like pranayama, meditation, and mindful breathing can help people become more psychologically flexible, which is a necessary quality that enables one to handle stress, control emotions, and face difficulties with more poise.

## Introduction

Athletes' tactical, technical, physiological, psychological, and social traits primarily influence their performance in sports. Sweat rate, blood pressure, heart rate, breathing rate, blood oxygen saturation, body temperature, serum levels of various stress hormones (like cortisol), as well as immunological functions (such as suppression of lymphocyte activation), are examples of physiological parameters that demonstrate adaptation to the new environment [1,2]. Studies have suggested that physical activity is influenced by athletes' psychological well-being [3]. Athletes' psychological or social traits that manifest in their athletic performance include stress management, motivation, mental acuity, and teamwork [4]. It is commonly known that playing sports can help people feel less stressed and anxious. However, because sports are competitive, they can negatively affect athletes' mental well-being and make them fearful of failing [5,6]. Stress can be both a mental and physical reaction to outside stimuli that exceed a person's capacity for behavioral adaptation and coping. Stressors are any cognitive or environmental conditions that cause stress [7]. Mental stress and conditions like anxiety, eating disorders, substance use disorders, depression, and psychological distress can be exacerbated by athletic culture [8,9]. Evidence indicates that better sleep is linked to reduced stress and anxiety, improved performance, and competitive success in athletes, in addition to the adaptive process [10,11]. There are several ways to improve the quality and pattern of an athlete's sleep, such as napping, sleep extension, post-exercise recovery techniques, and sleep hygiene [12]. To increase the likelihood of confidence building, traditional sports psychology teaches students how to minimize distractions and unwanted negativity.

However, research on lowering anxiety to improve sports performance has shown mixed results [13]. Acceptance and commitment therapy focuses on mindfulness as a means of bringing conscious attention in a curious, receptive way [14]. The primary goal of mindfulness practice is to remain open-minded and nonjudgmental about the circumstances at hand. Frequent practice enhances skills and directs focus toward stimuli that capture interest, thereby improving task performance [15]. Numerous studies have substantiated the mental and physical advantages of yoga. Mindfulness, yoga, and other behavioral therapies are associated with psychological balance. Yoga and other mind-body exercises that incorporate mindfulness emphasize developing awareness to listen to and respond to physical sensations. This awareness improves athletes' psychological health and physical performance in challenging circumstances [16,17]. The study aimed to assess the effects of yoga practice on athletes' psychological flexibility, stress levels, and mindfulness.

## Materials and methods

Research approach and design

A quantitative research approach was employed. The post-test-only, parallel-group, randomized controlled design was utilized for the study. It was an open-label trial, as blinding was not feasible due to the nature of the intervention. 

Sample size

For an effect size of 1.264, an alpha error of 0.05, and a power of 0.95, the sample size was estimated to be sufficient to compare the means of two independent groups. G*Power version 3.1 (Heinrich-Heine-Universität Düsseldorf, Düsseldorf, Germany) estimated that about 72 participants would be needed, and a 20% attrition rate was also considered. For this study, a final estimated sample size of 44 (in each group) was selected [18].

Inclusion and exclusion criteria

This was a single-center study. Participants were selected based on the inclusion criteria from the athletes studying at Banaras Hindu University, Varanasi, India. The study included individuals of both genders, aged between 18 and 45 years, who were recreational athletes participating in a variety of sports, including curling, volleyball, basketball, swimming, football, cricket, wrestling, badminton, skating, kabaddi, tennis, and hockey. To be eligible, participants had to be engaged in their respective sports.

**Figure 1:**
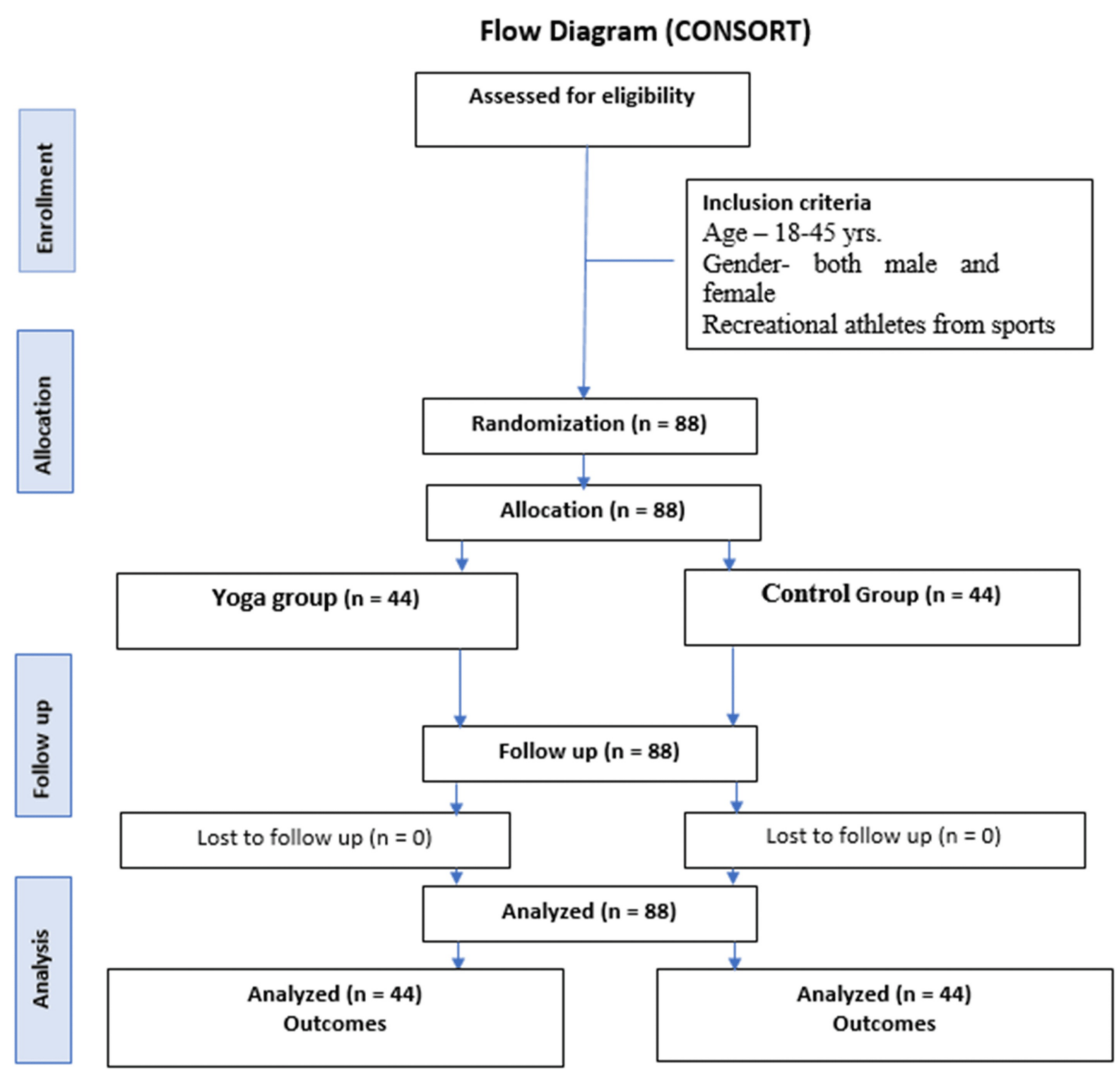
Flow diagram (CONSORT) CONSORT: Consolidated Standards of Reporting Trials

Individuals with a history of severe mental illnesses, such as psychosis, mania, bipolar disorders, or obsessive-compulsive disorder; chronic conditions, such as hypertension, type 2 diabetes, allergic asthma, or tuberculosis; and endocrine or metabolic diseases, such as hypothyroidism or hyperthyroidism, were excluded. Those with a history of substance dependence, physical disability, psychotropic drug use, or smoking were also excluded. Additionally, participants were ineligible if they were younger than 18 years, had previously practiced yoga or mindfulness training, or participated in less than four hours of weekly athletic training (Figure 1).

Yoga intervention

Over the course of a month (05/03/2024 to 03/04/2024), the yogic intervention comprised a planned practice of pranayama (breath control) and meditation, which started with a customary prayer. To improve physical vitality, mental clarity, and emotional balance, the practice included a variety of breathing techniques, including *Kapal Bhati*, *Ujjai*, *Bhastrika*, *Sitli*, *Sitkari*, *Anulom Vilom*, and *Bhramari*. Each technique was performed for a set amount of time for five days a week. To promote participants' general well-being, sessions ended with Om chanting and a peace prayer (Table 1) [19].

**Table 1:** Yoga intervention Transliteration and translation of the yogic intervention prayer: *Yogena chittasya padena vacha malam sharirasya cha vaidyakena. Yopakarottam pravaram muninam, patanjalim pranajaliranato'smi.* (Let us bow down to the greatest of sages, Patanjali, who gave yoga for peace and purity of mind, grammar for clarity and purity of speech, and medicine to remove the toxicity of the body for perfection of health.) Transliteration and translation of the Om chanting mantra: *Om sarve bhavantu sukhinah, sarve santu niraamayaah, sarve bhadraanni pashyantu. Maa kashcid-duhkha-bhaag-bhavet. Om shaantih shaantih shaantih. *(Om, may all be happy, may everyone be healthy, may all be well. Let no one be a part of the suffering. Om, peace, peace, peace.)

Yoga intervention	
Yogic intervention prayer: योगेन चित्तस्य पदेन वाचााााााां।मलाां शरीरस्य चा वैद्यकेन ॥ योऽपाकरोत्तमाां प्रवराां मनुीनााां। पतञ्जिलाां प्राञ्जिलरानतोऽचस्म ॥ (in Sanskrit)	
Skull cleansing (*Kapal Bhati*)	2-3 min
Heating pranayama (*Ujjai *and *Bhastrika*)	5-7 min
Cooling pranayama (*Sitli *and *Sitkari*)	5-7 min
Alternate nostril breathing (*Anulom Vilom*)	6 min
Bee breathing (*Bhramari*)	3-4 min
Meditation (Om chanting): ॐ सर्वे भवन्तु सुखिनः । सर्वे सन्तु निरामयाः । सर्वे भद्राणि पश्यन्तु । मा कश्चित् दुःख भाग्भवेत् ॥ ॐ शान्तिः शान्तिः शान्तिः॥ (in Sanskrit)	5-7 min

Outcome variables

Perceived Stress Scale (PSS)

A 10-item self-report survey, called the PSS, was used to gauge overall stress levels during the previous month. A five-point Likert scale, ranging from 0 to 4, was used to weigh the items. Higher perceived stress levels were reflected in higher scores. With Cronbach's alpha values of 0.75 to 0.91, the scale is a valid indicator of stress perception [20].

Mindfulness Attention Awareness Scale (MAAS)

The 15 reverse items make up the single-dimension self-report MAAS. It is also among the most widely used tools for measuring mindfulness. Each item was evaluated using a six-point Likert scale (where 1 stands for "almost always," 2 for "very often," 3 for "somewhat frequently," 4 for "somewhat infrequently," 5 for "very seldom," and 6 for "almost never"). The range of the average score is 15 to 90, with a lower average score indicating a decrease in mindfulness. The internal consistency reliability ranges from 0.72 to 0.81 [21].

Questionnaire on Acceptance and Action-II (AAQ-II)

This seven-item test evaluated experience avoidance and psychological rigidity. A seven-point scale was used, where 1 represents "never true" and 7 represents "always true" [22].

Randomization, allocation, and concealment

A total of 88 participants were recruited after screening and randomly allocated in a 1:1 ratio to the yoga group (n = 44) and control group (n = 44). The control group did not receive any intervention. Computer-generated randomization was employed and sequentially numbered opaque sealed envelopes were used to allocate concealment. Only the outcome assessors were blinded in this study, as blinding was impossible with the yoga and control groups; thus, participants could not be blinded. One of the investigators was the yoga trainer. Outcomes data were measured at pre- and post-intervention periods. 

Clinical trial registration and ethical approval

The trial was approved by the Institutional Ethics Committee (Dean/2023/EC/6126) and registered with India's clinical trial registry (CTRI/2024/02/063135).

Data collection

Following the research inclusion criteria, participants were contacted, and the yogic intervention was presented to them. Informed consent was obtained. The intervention included meditation and pranayama, which the intervention group engaged in for a month, while no treatment was given to the control group. Participants in the yoga groups were monitored throughout the intervention month.

Data extraction and analysis

On the first and 30th days, data extraction was conducted for both the yoga and control groups. Standard deviation, standard error, and mean were used to measure all quantitative variables. Frequencies and proportions for categorical variables were included. Every statistical test that was performed had a two-sided significance level of p < 0.05. Independent t-tests were utilized to investigate the differences within and between study groups. To determine whether the confounding variable had a significant impact, regression analysis was utilized.

## Results

Demographic details

A total of 88 participants were included in the study, and they were split equally between the two groups: control (44 participants) and yoga (44 participants). There were 32 (72.7%) men and 12 (27.3%) women in the yoga group, compared to 34 (77.3%) men and 10 (22.7%) women in the control group. In terms of eating patterns, 20 (45.5%) participants in the yoga group were vegetarians, compared to 14 (31.8%) in the control group. The remaining participants in both groups were not vegetarians (Table 2).

**Table 2:** Demographic details of the participants

Category	Yoga group, N (%)	Control group, N (%)
Gender		
Men	32 (72.7%)	34 (77.3%)
Women	12 (27.3%)	10 (22.7%)
Eating pattern		
Vegetarian	20 (45.5%)	14 (31.8%)
Non-vegetarian	24 (54.5%)	30 (68.2%)
Athletic level		
International	2 (4.5%)	0 (0%)
National	22 (50.0%)	12 (27%)
State	11 (22%)	18 (41%)
University	11 (23.5%)	14 (32%)
History of metabolic disease		
Yes	5 (11.4%)	4 (9.1%)
No	39 (88.6%)	40 (90.9%)
Family history of metabolic disease		
Yes	4 (9.1%)	4 (9.1%)
No	40 (90.9%)	40 (90.9%)
Smoking/alcohol use		
Yes	10 (22.7%)	6 (13.6%)
No	32 (72.7%)	38 (86.4%)
Prefer not to say	2 (4.5%)	0 (0%)

The yoga group had two athletes competing at the international level and a larger national representation in terms of athletic experience and competition level (22 (50%) participants). The control group, on the other hand, had none at the international level but more at the state (18 participants (40.9%)) and university (14 (31.8%)) levels. A small percentage of participants in both groups reported having between six months and two years of athletic experience, while the majority - 38 (86.4%) participants in the yoga group and 40 (90.9%) participants in the control group - reported having more than two years of experience.

In terms of health-related factors, four participants in the control group and five in the yoga group reported having a history of metabolic disease, while the remaining participants in both groups did not have any such conditions. Likewise, four individuals from each group had a family history of metabolic disease. Ten participants in the yoga group and six in the control group said "yes" when asked if they smoked or drank alcohol. Furthermore, two participants in the yoga group chose not to reveal their history of smoking or alcohol consumption, whereas 32 (72.7%) participants in the yoga group and 38 (86.4%) participants in the control group reported no such history.

The study's comparability is supported by this demographic breakdown, which shows a fairly balanced distribution between the two groups in terms of gender, eating habits, athletic experience, health status, and lifestyle factors.

Stress, mindfulness, and psychological flexibility

The yoga group's mean score on the MAAS, which measures mindfulness, was 35.59 (standard deviation (SD) = 3.05), while the control group's mean score was 36.05 (SD = 2.28). These findings imply that, before the intervention, both groups had comparable levels of present-moment awareness. The AAQ, which measures psychological flexibility and acceptance, revealed a somewhat lower mean in the yoga group (35.23, SD = 4.82) than in the control group (37.64, SD = 4.44). This suggests that initially, members of the control group might have exhibited slightly greater psychological flexibility (Table 3).

**Table 3:** Participants baseline mean values of stress, mindfulness, acceptance, and action scores AAQ: Acceptance and Action Questionnaire; MAAS: Mindfulness Attention Awareness Scale; PSS: Perceived Stress Scale

Parameter	Group	Mean	Standard deviation
PSS	Yoga	31.5455	3.75090
	Control	31.1364	3.77076
MAAS	Yoga	35.5909	3.04973
	Control	36.0455	2.27779
AAQ	Yoga	35.2273	4.81992
	Control	37.6364	4.43520

Table 4 presents the post-intervention scores of stress, mindfulness, acceptance, and action outcomes variables. It can be seen that participants' stress levels were significantly reduced after the intervention, and the mean score was also lower than that of the control group (p < 0.001). Participants in the yoga group had lower mean scores compared to the control group (p < 0.001). Yoga also improved their mindfulness and awareness; scores were significantly higher than those of the control group. Yoga participants reported higher levels of dispositional mindfulness and lower reported negative emotional states (p < 0.001). Furthermore, yoga participants reported improved psychological flexibility in daily life; their average scores were lower than those in the control group (p < 0.001) (Table 4).

**Table 4:** Differences between both groups concerning the mean values of stress, mindfulness, acceptance, and action scores Independent t-test applied; p-value significant at < 0.05. (*) represents a significant p-value. AAQ: Acceptance and Action Questionnaire; MAAS: Mindfulness Attention Awareness Scale; PSS: Perceived Stress Scale

Parameter	t-value	p-value	Mean difference	95% confidence interval of the difference	
				Lower	Upper
PSS	-10.755	0.002*	-11.50000	-13.65788	-9.34212
MAAS	13.783	0.011*	24.77273	21.14561	28.39985
AAQ	-9.288	0.002*	-11.45455	-13.94331	-8.96579

Table 5 suggests that stress alleviation (adjusted R^2^ = 0.660, p = 0.000), followed by improving mindfulness (adjusted R^2 ^= 0.581, p = 0.000), could be strong predictors for enhancing psychological flexibility skills in athletes' daily life. Conclusively, higher mindfulness (as measured by MAAS) is associated with a decrease in psychological rigidity - possibly related to stress, anxiety, or other distress indicators (Table 5). 

**Table 5:** Effect of stress and mindfulness on participants psychological flexibility (regression coefficient) Dependent variable: AAQ. (*) represents a significant p-value. AAQ: Acceptance and Action Questionnaire; MAAS: Mindfulness Attention Awareness Scale; PSS: Perceived Stress Scale

Model	Unstandardized coefficients		Standardized coefficients		t	Significance
	B	Standard error	Beta	Adjusted R^2^		
PSS	0.85	0.092	0.817	0.66	9.194	0.000*
MAAS	-0.392	0.05	0.768	0.581	-7.783	0.000*

## Discussion

The purpose of the study was to evaluate how yoga interventions affect stress, mindfulness, and awareness of athletes in their day-to-day lives. Participants in yoga groups reported considerably less stress according to the results. Additionally, yoga improved their acceptance, action, mindfulness, and awareness in day-to-day activities. Participants' psychological flexibility in daily life is also linked to their stress levels and dispositional mindfulness. Yoga and meditation can lessen stress and anxiety by influencing the limbic system and the brain's chemical gamma-aminobutyric acid (GABA) [23]. Acute changes in hormone levels can have either a beneficial or detrimental effect on an athlete's performance. This variability is closely associated with acute changes in hormone levels, particularly the release of stress-related hormones such as cortisol and adrenaline. These hormonal changes impact physiological arousal, cognitive appraisal processes, and athletes' capacity to perform under pressure [24]. This study's findings align with those of other investigations [25-30]. An analysis conducted by a multidisciplinary team has provided essential insights into the advantages and complementary effects of yoga and mindfulness meditation on athletes' mental well-being and physical performance [31].

According to earlier research, the yoga group's mindfulness scores were significantly higher than those of the control group. The findings suggest that yoga interventions, in addition to enhancing physical functioning patterns and mindfulness levels, may have a positive effect on sports injuries [32]. A previous study indicated that the yoga group's MAAS scores significantly improved following the intervention compared to the control group, indicating greater mental awareness and consistency with expected outcomes. Following yoga interventions, athletes have also shown increased goal-directed energy and improved mindfulness according to studies on sports teams [25]. For a month, the experimental group received the intervention twice a week for 30 minutes each time. In terms of their self-confidence scores at the second and third time points, this study revealed a significant difference between the dimension groups. A logistic regression analysis revealed that the intervention had a beneficial impact on improving athletic performance [33]. According to the study, basketball players' performance was significantly enhanced by the mindfulness technique. Additionally, mindfulness lessened sensory avoidance in athletes, with a moderate amount of difference observed between the groups. In line with other research findings, this study discovered that athletes were less likely to accept negative thoughts and feelings [34-37]. The results of the current randomized controlled trial consistently support the positive effects of a month-long yoga practice on athletes' performance and mental well-being.

The study's novelty and implications

This study is unique in that it assessed yoga practice's effects on athletes' stress, mindfulness, and psychological flexibility all at once - an approach that isn't often covered in a single study. The study also determined the most powerful psychological predictor impacted by yoga practice, adding significant knowledge to the existing body of literature. The results indicate that yoga is a therapeutic approach that is both cost-effective and supportive, and it can be used in a variety of settings. Beyond its immediate psychological advantages, yoga can be used as a self-directed behavioral intervention to enhance athletes' resilience, emotional control, and overall well-being. Yoga practice also boosts self-efficacy and self-confidence, making it a useful adjunctive tactic for encouraging the holistic development of young athletes.

Strengths and limitations

Because this was a randomized controlled trial, the results' internal validity was preserved by the control group and randomization. The determined sample size was ideal for analyzing the results. Strong predictors for enhancing psychological flexibility skills in athletes' daily lives were examined using regression analysis. However, due to the nature of the intervention, blinding was not feasible in this open-label trial. The validity of the results was impacted by the use of subjective questionnaires for measurement and comparison. The variables that were evaluated were self-reported. The results might have been affected by variations in the athletes' experiences. Furthermore, it was a single-center study, and follow-up evaluations over time were not included.

## Conclusions

According to the study's findings, athletes' dispositional mindfulness and stress levels can be considerably improved by practicing yoga regularly for a month. Due to their rigorous training regimens, competitive pressure, and requirement for consistent performance, athletes especially need these improvements. Yoga provides a comprehensive strategy for stress management and mental clarity by combining breathing exercises, meditation, and mindful awareness. Improved mindfulness enables athletes to respond more calmly under pressure, better handle distractions, and maintain present-moment focus. Additionally, it has been demonstrated that yoga promotes the growth of psychological flexibility, a critical quality for overall mental health and athletic performance. Psychological flexibility is the capacity to adapt to changing conditions and deal with setbacks. Beyond these psychological benefits, yoga helps athletes develop mental resilience, equipping them with the skills needed to bounce back more quickly from setbacks, injuries, or stress. Furthermore, yoga practice fosters self-regulation, self-awareness, and a sense of control, teaching behavioral strategies that can be empowering in both daily life and athletics. According to research, yoga is a beneficial, cost-effective, and easily accessible intervention that can be integrated into athletes' training regimens to support mental health, improve performance, and promote long-term psychological development.
